# Metaproteomic Insights Into the Microbial Community in Pozol

**DOI:** 10.3389/fnut.2021.714814

**Published:** 2021-08-20

**Authors:** Jocelin Rizo, Daniel Guillén, Gloria Díaz-Ruiz, Carmen Wacher, Sergio Encarnación, Sergio Sánchez, Romina Rodríguez-Sanoja

**Affiliations:** ^1^Departamento de Biología Molecular y Biotecnología, Instituto de Investigaciones Biomédicas, Universidad Nacional Autónoma de México, Mexico City, Mexico; ^2^Programa de Doctorado en Ciencias Biológicas, Universidad Nacional Autónoma de México, Mexico City, Mexico; ^3^Departamento de Alimentos y Biotecnología, Facultad de Química, Universidad Nacional Autónoma de México, Mexico City, Mexico; ^4^Departamento de Genómica Funcional de Procariontes, Centro de Ciencias Genómicas, Universidad Nacional Autónoma de México, Cuernavaca, Mexico

**Keywords:** fermented food, food metaproteomics, traditional fermented food, pozol, lactic acid bacteria, *Streptococcus*

## Abstract

Pozol is an acidic, refreshing, and non-alcoholic traditional Mayan beverage made with nixtamalized corn dough that is fermented spontaneously. The extensive analysis of the microbiology, biochemistry and metaproteomics of pozol allowed the construction of a comprehensive image of the fermentation system. The main changes in both the substrate and the microbiota occurred in the first 9 h of fermentation. The increase in microorganisms correlated with the drop in pH and with the decrease in the contents of carbohydrates, lipids, and nitrogen, which shows that this stage has the highest metabolic activity. Bacterial proteins were mainly represented by those of lactic acid bacteria, and among them, the proteins from genus *Streptococcus* was overwhelmingly the most abundant. Yeast proteins were present in all the analyzed samples, while proteins from filamentous fungi increased up to 48 h. The metaproteomic approach allowed us to identify several previously unknown enzyme complexes in the system. Additionally, enzymes for hydrolysis of starch, hemicellulose and cellulose were found, indicating that all these substrates can be used as a carbon source by the microbiota. Finally, enzymes related to the production of essential intermediates involved in the synthesis of organic acids, acetoin, butanediol, fatty acids and amino acids important for the generation of compounds that contribute to the sensorial quality of pozol, were found.

## Introduction

Pozol is a traditional refreshing, acidic and nonalcoholic beverage made from fermented nixtamalized maize dough. It is consumed in Southeastern Mexico, in the Mayan region, not only as an important part of the diet but also as a culturally important mainstay in traditional medicine and ceremonies (1, 2). Pozol preparation begins with the careful selection of maize grains to remove any contaminants. Then, the grains are cooked in water with lime by a process known as nixtamalization; the kernels are subsequently washed to remove the lime. The cooked grains are coarsely ground, and the resulting heterogeneous dough is kneaded and molded to form a compact ball that is wrapped in banana plant leaves and left at ambient temperature to allow spontaneous fermentation from just a few hours to several days. The fermented dough is then suspended in water and consumed daily as a refreshing beverage (1, 3). After nixtamalization, the soluble sugars present in maize drastically decrease, so it is logical to think that the main carbon source used during fermentation is starch.

More than 40 different species of bacteria, fungi and yeast have been reported from pozol (4–7). Most microbiological studies have focused on the isolation and identification of bacteria, mainly lactic acid bacteria (LAB), since LAB are the predominant group during fermentation and because of their relevance as probiotics in fermented foods and in the industry as starter cultures (4–9). In contrast, other bacteria, fungi and yeast have been poorly studied, and the ecological niche of microorganisms in pozol fermentation has not been reported, generating a partial understanding of the fermentation mechanism.

Elucidation of the role and behavior of microorganisms in different microbial ecosystems can be addressed with different nonexclusive approaches that target different types of molecules. A powerful strategy to understand the functions of fermentation microbiota is protein study by metaproteomic techniques, which aim to identify “metagenome” expression patterns under different conditions and allow an accurate picture of the metabolic processes that actually occur in microorganisms (10).

Metaproteomics has been successfully applied in different food fermentations to describe not only the microbial composition and succession but also, its role in the process and the relationship of microorganisms with flavor development (11–14). In this study, a metaproteomic approach was used for a detailed analysis of the pozol fermentation, to infer from the changes observed in proteins, the functional role of the microbiota during fermentation.

## Materials and Methods

### Samples

Freshly ground nixtamal dough samples were acquired from two different producers at Pino Suárez market in Tabasco, México. The samples were mixed until homogeneous, and 300 g balls were wrapped in sanitized banana leaves and incubated at 37°C. Triplicate fermentations were performed, and samples were taken for analysis at 0, 9, 24 and 48 h. Collected samples were stored at −80°C for further metaproteomic analyses. Microbiological and chemical studies were carried out immediately after the fermentation time had elapsed.

### Enumeration of Microorganisms

Serial dilutions of 25 g homogenized dough sample in 225 mL of 0.1% (w/v) peptone water (Bacteriological peptone, Oxoid L37) were used for microbial enumerations with the following media: plate count agar (Oxoid CM 325) for total aerobic mesophilic bacteria, MRS agar (BD Difco) for LAB, MRS starch (containing 1% soluble starch instead of glucose) for amylolytic lactic acid bacteria (ALAB), violet red bile glucose agar for enterobacteria (Oxoid CM 485) and potato dextrose agar (Oxoid CM 139) acidified to pH 3.5 for fungi (3, 4, 7). Microbial enumerations were done in triplicate.

### Analytical Methods

#### pH

For this purpose, dough samples (1 g) were homogenized in a mortar and mixed with 5 mL distilled water. The pH was determined using an OakTon pH meter with a reference glass electrode.

#### Proximate Composition of Pozol

Dough samples (10 g) were pulverized in a mortar with solid CO_2_ until powder was obtained. Lipid, fiber and nitrogen-free extract (NFE) contents were determined as described previously (15). Total soluble carbohydrates (those solubilized by dissolving the pozol powder in water) were determined by the phenol-sulfuric acid method (16).

All determinations were performed in triplicate and analyzed with one-way analysis variance (ANOVA) using GraphPad Prism 4 software. Significant differences were estimated with the *post hoc* Tukey test.

### Protein Extraction and Quantification

Protein extraction was performed in triplicate for each fermentation time using a method that combines the methodological approaches as previously reported (17, 18). First, a representative pozol sample containing inner, middle and external portions of the dough was ground with solid CO_2_ until a powder was obtained. Then, 1 g of sample was mechanically mixed at room temperature for 2 h with 10 mL of 0.1 M citrate-phosphate buffer pH 5 with protease inhibitor 1:100 (Sigma Protease Inhibitor cocktail for general use). The samples were centrifuged (800 × g for 10 min) at 4°C, and the supernatant was collected. Two more extractions were performed on the obtained pellet with 10 mL of buffer and mechanical agitation for 30 min. The resulting supernatant was cloth filtered and centrifuged (13,200 × g for 10 min) at 4°C. The pellet was used for protein extraction as described previously (18); briefly, the pellet was treated with 1.0 mL of buffer ASB-14 (7 M urea, 2 M thiourea, 2% CHAPS (w/v), 2% ASB-14 (w/v), and 30 mM buffer Tris-HCl pH 7) and glass beads (Sigma 710-1180 μm) were added to the pellet. The samples were vortexed vigorously for 5 min each 20 min for 1 h at room temperature and then centrifuged three times for 10 min at 13,200 × g and 4°C. The clarified supernatant from each centrifugation was recovered. Three extractions were made per fermentation time.

After the extraction, the protein concentration was quantified using the Bio-Rad Protein Assay method (Bio-Rad), and bovine serum albumin (BSA) was used as the standard. To avoid any interference related to the extraction buffer, samples were precipitated with 10% (w/v) trichloroacetic acid (TCA) (19), solubilized in 50 μl of 6 M urea and finally dissolved in 750 μl of distilled water.

### 1D-GE, Trypsin Digestion and LC–MS/MS

Protein from the supernatant (50 μg) was precipitated with 10% (w/v) TCA and collected by centrifugation (13 200 × g, 20 min, 4°C). The pellets were resuspended in 10 μL of 6 M urea and 10 μL of 2 × Laemmli buffer and heated 5 min at 96°C for. The samples were resolved on SDS-PAGE 10% acrylamide using 10 mA per gel until the dye migrated off the gel. The gel was stained with Coomassie brilliant blue, and each lane was divided into 6 pieces and sent to Institut de Recherches Cliniques de Montréal (IRCM) for sequencing.

#### Protein Digestion With Trypsin

Both digestion and LC-MS/MS analysis were performed as previously described (18). Gel pieces were washed with water for 5 min and destained twice with destaining buffer (100 mM sodium thiosulfate, 30 mM potassium ferricyanide) for 15 min. An additional wash of 5 min was performed after destaining with a buffer of ammonium bicarbonate (50 mM). Gel pieces were then dehydrated with acetonitrile. Proteins were reduced with reduction buffer (10 mM dithiothreitol, 100 mM ammonium bicarbonate) for 30 min at 40°C and then alkylated with alkylation buffer (55 mM iodoacetamide, 100 mM ammonium bicarbonate) for 20 min at 40°C. Gel pieces were dehydrated and washed at 40°C with acetonitrile (ACN) for 5 min before discarding all the reagents. Gel pieces were dried for 5 min at 40°C and then rehydrated at 4°C for 40 min with trypsin solution (6 ng/μL trypsin sequencing grade from Promega and 25 mM ammonium bicarbonate). The trypsin concentration was kept low to reduce signal suppression effects and background originating from autolysis products when performing LC–MS/MS analysis. Protein digestion was performed at 58°C for 1 h and stopped with 15 μL of 1% formic acid/2% acetonitrile. The supernatant was transferred to a 96-well plate, and peptide extraction was performed with two 30-min extraction steps at room temperature using extraction buffer (1% formic acid/50% ACN). All peptide extracts were pooled into 96-well plates and then thoroughly dried in a vacuum centrifuge. The plates were sealed and stored at – 20°C until LC–MS/MS analysis.

#### LC–MS/MS

Prior to LC–MS/MS, peptide extracts were resolubilized under agitation for 15 min in 12 μL of 0.2% formic acid and then centrifuged at 2,000 × g for 1 min. The LC column was a C18 reversed-phase column packed with a high-pressure packing cell. A 100-mm-long 75 μm i.d. fused silica capillary was packed with a C18 Jupiter 5 μm 300 Å reverse-phase material (Phenomenex). This column was installed on the nanoLC-2D system (Eksigent) and coupled to the LTQ Orbitrap Velos (Thermo Fisher Scientific). The buffers used for chromatography were 0.2% formic acid (buffer A) and 100% acetonitrile/0.2% formic acid (buffer B). During the first 12 min, 5 μL of sample was loaded on the column with a flow of 650 nL/min, and subsequently, the gradient went from 2 to 80% buffer B in 20 min and then returned to 2% buffer B for 10 min. LC–MS/MS data acquisition was accomplished using an eleven-scan event cycle comprised of a full scan MS for scan event one acquired in the Orbitrap, which enables high resolution/high mass accuracy analysis. The mass resolution for MS was set to 60,000 (at m/z 400) and used to trigger the ten additional MS/MS events acquired in parallel in the linear ion trap for the top three most intense ions. The mass over charge ratio range was from 380 to 2000 for MS scanning with a target value of 1,000,000 charges and from ~ 1/3 of the parent m/z ratio to 2000 for MS/MS scanning with a target value of 10,000 charges.

The data-dependent scan events used a maximum ion fill time of 100 ms and 1 microscan to increase the duty cycle for ion detection. Target ions already selected for MS/MS were dynamically excluded for 15 s. Nanospray, capillary and tube lens voltages were set to 0.9–1.6 kV, 5 and 100 V, respectively. Capillary temperature was set to 225°C. MS/MS conditions were as follows: normalized collision energy, 35 V; activation q, 0.25; and activation time, 30 ms.

#### Database Searching

Raw MS files were analyzed by Mascot (Matrix Science, London, UK; version 2.5.1) and X! Tandem (The GPM, thegpm.org; version 2007.01.01.1) or by MaxQuant (version 1.6.2.10) with the default parameters (20).

Tandem mass spectra were first analyzed by Mascot version 2.5.1. Charge state deconvolution and deisotoping were not performed. Mascot was set up to search the UniProt sprot database (selected for bacteria, unknown version, 329683 entries and for fungi, unknown version, 31499 entries), UniProt_Maize_txid4577_20160927 database (unknown version, 160789 entries) and UniProt_Archaea_txid2157_20160921 database (unknown version, 1351908 entries) with trypsin as the digestion enzyme. O+18 of pyrrolysine and carbamidomethyl of cysteine were specified in Mascot as fixed modifications. Oxidation of methionine was specified in Mascot as a variable modification.

For the analysis in MaxQuant software, the identification of individual peptides was conducted in protein groups. The MaxQuant searches were executed against the CAZy database (CAZyDB.07312019.fa). The following search parameters were used: enzymatic cleavage rule of Trypsin/P and a maximum of two missed cleavage sites, carbamidomethylation on cysteine as fixed modification, whereas protein N-terminal acetylation and methionine oxidation were defined as variable modifications for the database searches. All entries were filtered using a false positive rate of 1%, and all false positives were removed.

For all analyses, the first search peptide tolerance was 20 ppm, and the main search peptide tolerance was 4.5 ppm. A fragment ion mass tolerance of 0.60 Da and a parent ion tolerance of 10 ppm were established.

### Protein Identification Criteria

Scaffold_4.8.6 (Proteome Software Inc., Portland, OR) was used to validate MS/MS-based peptide and protein identifications for Mascot and X! Tandem results. For the validation of the identified proteins, the FDR was adjusted to obtain probabilities >95% for the peptides and 99% for the proteins. Both probabilities were assigned by the Protein Prophet algorithm (21). Proteins that contained similar peptides and could not be differentiated based on MS/MS analysis alone were grouped to satisfy parsimony principles (Institut de Recherches Cliniques of Montreal, IRCM, Quebec, Canada). For the description of the microbial diversity, proteins that contained at least two identified peptides were considered, while for the functional classification, proteins that contained at least a unique peptide were accepted. In both cases, only the proteins that were identified in at least two of the three experimental replicates were considered, and false positives were manually removed.

## Results

### Enumeration of Microorganisms

**Figure 1:**
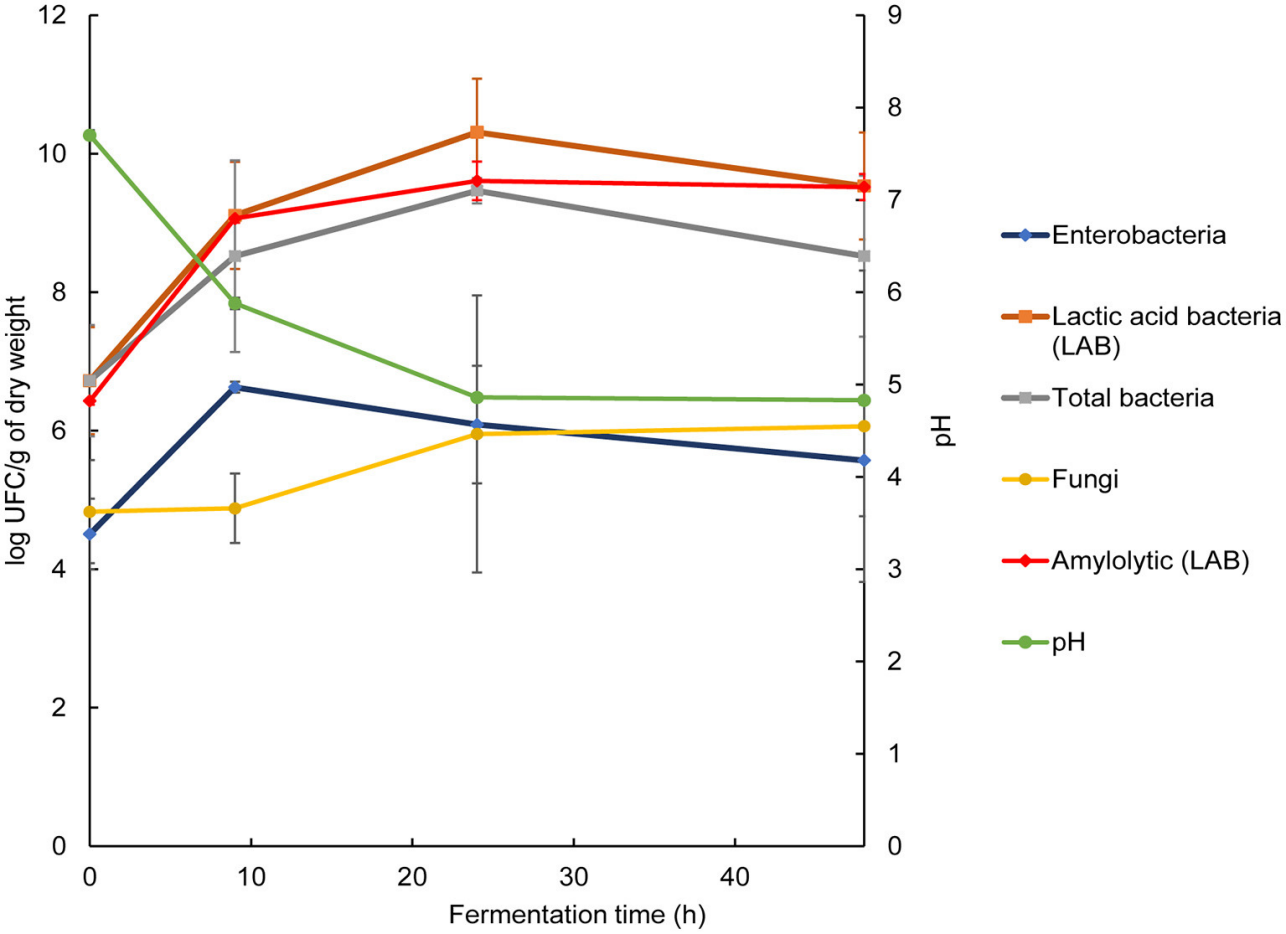
Microbial and chemical changes during pozol fermentation. Concentration of microorganisms and pH values. The results are expressed as the mean of three replicates ± standard deviation.

To correlate the metaproteomic data with the microbiota, the main microbial groups were quantified. We observed that just after nixtamalization, the bacterial counts for all groups analyzed were on the order of 10^6^ CFU, except for Enterobacteriaceae, which were at a concentration of 10^4^ CFU. Aerobic mesophiles began fermentation with a concentration of 10^6^ CFU · g of dough^−1^, increasing after 24 h of fermentation to 10^9^ CFU · g^−1^. Among them, LAB, the predominant group during fermentation, started with a count of 10^6^ CFU · g^−1^ and increased after 24 h of fermentation to 10^10^ CFU · g^−1^. This increase in bacterial count clearly coincided with the drop in pH by almost 3 units, from 7.75 to 4.83 (Figure 1).

Since starch is the main carbohydrate in nixtamal and LAB the most abundant microorganisms, previous studies considered that amylolytic lactic acid bacteria (ALAB) should play a relevant role during fermentation, providing glucose and maltooligosaccharides for the nonamylolytic microbial community (4, 9). ALAB started fermentation at a concentration of 10^6^ CFU · g^−1^, with an increase during the first 9 h of two logarithmic units (Figure 1).

Enterobacteriaceae are consistently found in traditional fermented foods. Their presence is generally an indicator of food quality and safety, and they are considered undesirable microorganisms. The microbial analysis revealed an initial count of 10^4^ CFU · g^−1^; during the first 9 h, the concentration increased to 10^6^ CFU · g^−1^, from which point the Enterobacteriaceae arrested their growth and began to decline slowly to 10^5^ CFU · g^−1^ after 48 h. On the other hand, as the pH value of pozol became more acidic, the fungal concentration increased, with a maximum of 10^6^ CFU · g^−1^ at 48 h (Figure 1).

At the same time, the carbohydrate content decreased, with the reduction in fiber content being the most obvious and interesting change since it involves the consumption of polysaccharides such as cellulose and hemicellulose (Table 1).

**Table 1:** Chemical composition of pozol at different fermentation times.

**Fermentation time (h)**	**Content in pozol (mg/g of dry dough)**			
	**Nitrogen-free extract**	**Total soluble carbohydrates**	**Crude fiber**	**Lipids**
0	845 ± 9.9A	12.22 ± 0.01A	24 ± 0.41A	46 ± 0.42A
9	839 ± 8A	11.67± 0.21AB	12 ± 0.05B	57 ± 0.12AB
24	841 ± 3A	12.66± 0.05B	9.0 ± 1B	64 ± 0.03B
48	832 ± 7A	12.96± 0.09C	8.0 ± 1B	68 ± 0.03C

*The results are expressed as the mean of three replicates ± standard deviation. Differences were evaluated by ANOVA followed by Tukey's multicomparison test (p < 0.05). Different uppercase letters indicate statistically significant differences between samples*.

### Metaproteomic Analysis

#### Microbial Proteins in Pozol Fermentation

The metaproteomic analysis at different fermentation times allowed the identification of 3215 proteins. The protein diversity analysis showed that eukaryotic, bacterial and archaeal domains were present during pozol fermentation, with abundances of 69.9, 28.5, and 1.6%, respectively. The protein analysis showed that the highest number of identified proteins belonged to maize (1,037 proteins).

**Figure 2:**
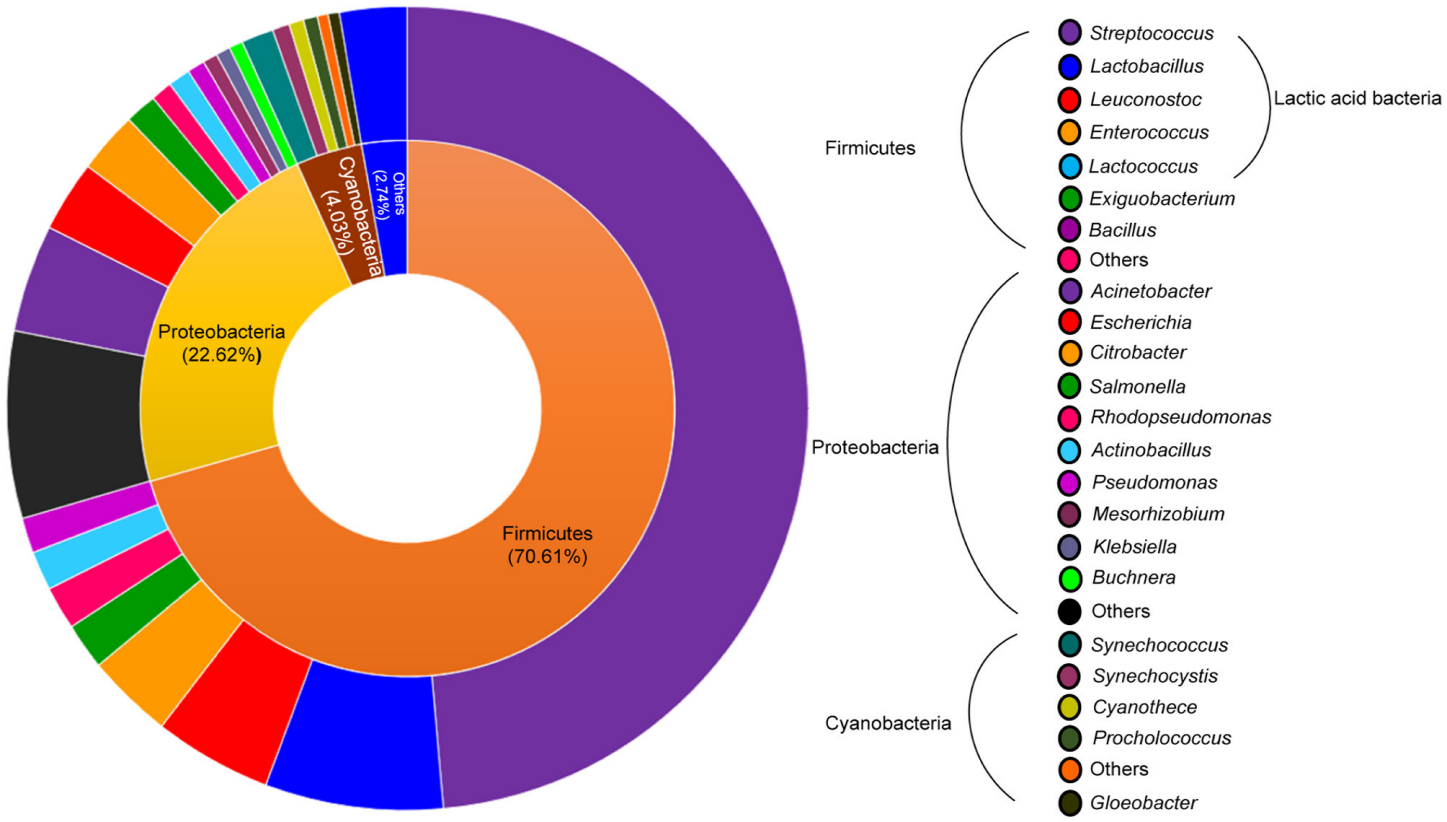
Donut plots representing the bacterial population in all pozol fermentation process. The inner and outer circles show the percentage distribution of the proteins at phylum and genus level, respectively.

To estimate the relative abundance of the microorganisms during the fermentation, the total number of identified proteins with two unique peptides was considered. In bacteria, a total of 704 proteins were identified, distributed in 8 different phyla and 71 different genera (Figure 2). The phylum classification showed that *Firmicutes* (70.61%) and *Proteobacteria* (22.62%) proteins were predominant. Other phyla accounted for <2% of the relative abundance and included *Actinobacteria, Chloroflexi, Spirochaetes, Planctomycetes* and *Bacteroidetes* proteins. The *Firmicutes* phylum was mainly represented by proteins of LAB (64.63%). *Streptococcus* was the prevalent genus in this community, with 47.87%, followed by the *Lactobacillus* genus (7.1%). Additionally, proteins of *Leuconostoc* (4.69%), *Enterococcus* (3.41%) and *Lactococcus* (1.56%) were identified but in minor abundance (Figure 2).

In the *Proteobacteria* phylum, proteins from enterobacteria were identified, with the *Escherichia* and *Citrobacter* genera as the most abundant (2.84 and 2.41%, respectively). Furthermore, proteins from nitrogen-fixing bacteria such as *Klebsiella* and *Enterobacter* were found. Other genera represented <1% of the metaproteome, i.e., *Actinobacillus, Staphylococcus*, and *Rhizobium*, among others.

**Figure 3:**
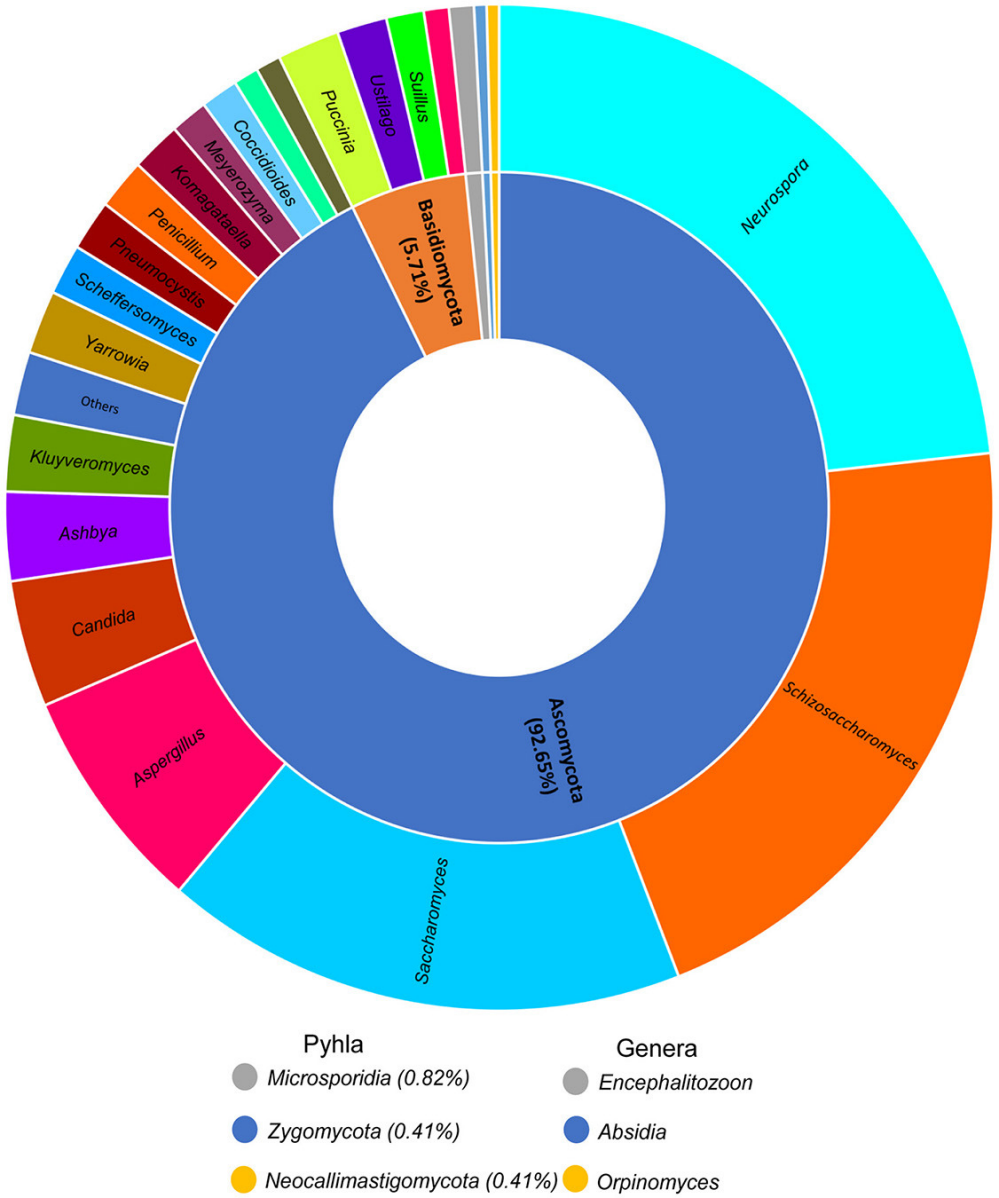
Donut plots representing the fungi population in all pozol fermentation process. The inner and outer circles show the percentage distribution of the proteins at phylum and genus level, respectively.

A lower number of fungal proteins were identified (245); these proteins were distributed in 5 phyla and 28 genera. Most of the proteins belong to the phylum *Ascomycota*, with the most abundant genera being *Neurospora* (23.36%), *Schizosaccharomyces* (20.90%) and *Saccharomyces* (17.21%). *Aspergillus* and *Candida* proteins represented 6.96 and 4.09% of the relative abundance, respectively. Other identified proteins belong to the genera *Ashbya, Kluyveromyces, Puccinia* and *Penicillium* (Figure 3).

Metaproteomic analysis allowed the identification of bacterial and fungal proteins whose genera have not been previously described in pozol, such as *Actinobacillus, Corynebacterium, Gloeobacter, Nitrobacter, Pantoea, Ajellomyces, Puccinia, Fusarium, Schizosaccharomyces* and *Yarrowia*, which represented <1% of the metaproteome.

#### Dynamic Changes During Pozol Fermentation

**Figure 4:**
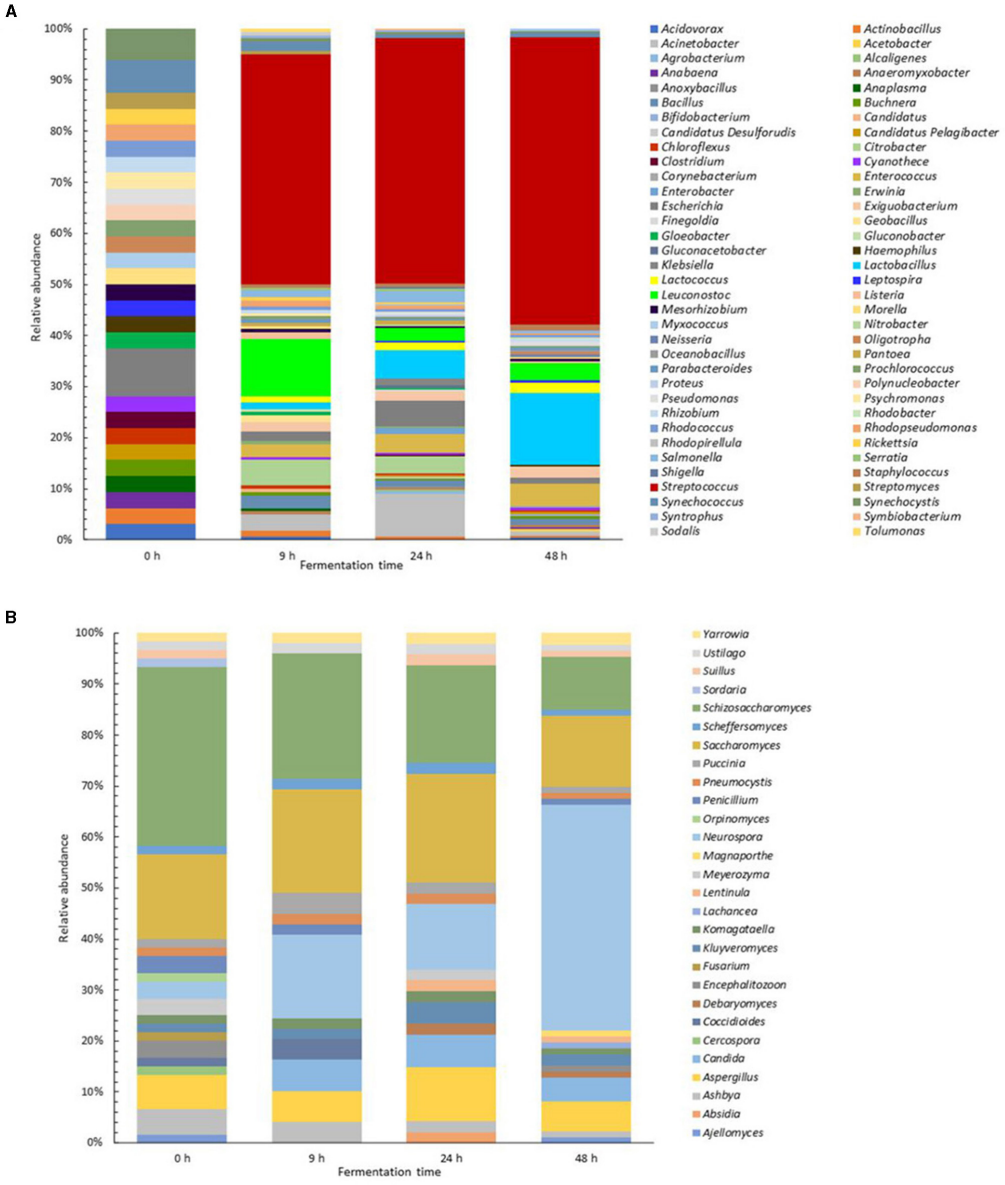
Abundance by gender and fermentation time of the proteins identified in the pozol metaproteome. Proteins were identified from 2 unique peptides in MS/MS analysis. **(A)** Bacteria proteins. **(B)** Fungal proteins.

The genus-level classification of the bacterial and fungal protein sequences showed the dynamics in pozol at different fermentation times. In the unfermented dough, a lower number of proteins were found, these proteins basically belong to environmental microorganisms, especially aquatic bacteria and cyanobacteria. Proteins were also found from endosymbionts and plant pathogens, soil bacteria, and proteins from animal commensals and pathogens to a lesser extent. Throughout fermentation, some of the detected proteins persisted, and the vast majority disappeared, indicating that the most abundant microorganisms at the beginning of fermentation were unable to grow in the dough. One exception to this was proteins of the genus *Escherichia*, a ubiquitous enterobacteria whose proteins were maintained during all the sampling times (Figure 4A).

Remarkably, during the first 9 h of fermentation, the proteins of LAB increased rapidly, reaching 64.63% of the total bacterial metaproteome. *Streptococcus* proteins were the most represented accounting for 45 and 48% at 9 h and 24 h, respectively. In the 9 h fermented sample, proteins from *Leuconostoc* (11%), *Citrobacter* (5%) and *Acinetobacter* (3%) were also present, but as fermentation progressed, *Acinetobacter* proteins were enriched, while *Citrobacter* and *Leuconostoc* proteins decreased to 3%. At this point, *Lactobacillus* proteins began to increase, reaching a relative abundance of 6%. At 48 h of fermentation, 79% of the identified proteins were from LAB, with absolute dominance of *Streptococcus* proteins, which reached 56% of the identified proteins. Proteins from the *Lactobacillus, Enterococcus* and *Lactococcus* genera were also detected, with relative abundances of 14, 4, and 2%, respectively, while *Leuconostoc* remained at 3%. *Enterobacteriaceae* proteins were identified at all fermentation times, but as fermentation progressed, their presence was reduced and, in the end, these proteins represented <3% of the metaproteome (Figure 4A).

Fungi are known to grow in pozol as well; however, they have not been practically studied. In this work, we observed that the predominant fungal proteins were from *Schizosaccharomyces* during the first 9 h, followed by *Saccharomyces*, which represented 21% of the metaproteome at 24 h of fermentation. The *Candida* genus was detected after 9 h, but the sample fermented for 48 h contained mostly proteins of the genus *Neurospora*, these proteins accounted for 44%; *Schizosaccharomyces* and *Saccharomyces* were reduced to <10% at this time (Figure 4B).

#### Functional Classification of the Metaproteome

**Figure 5:**
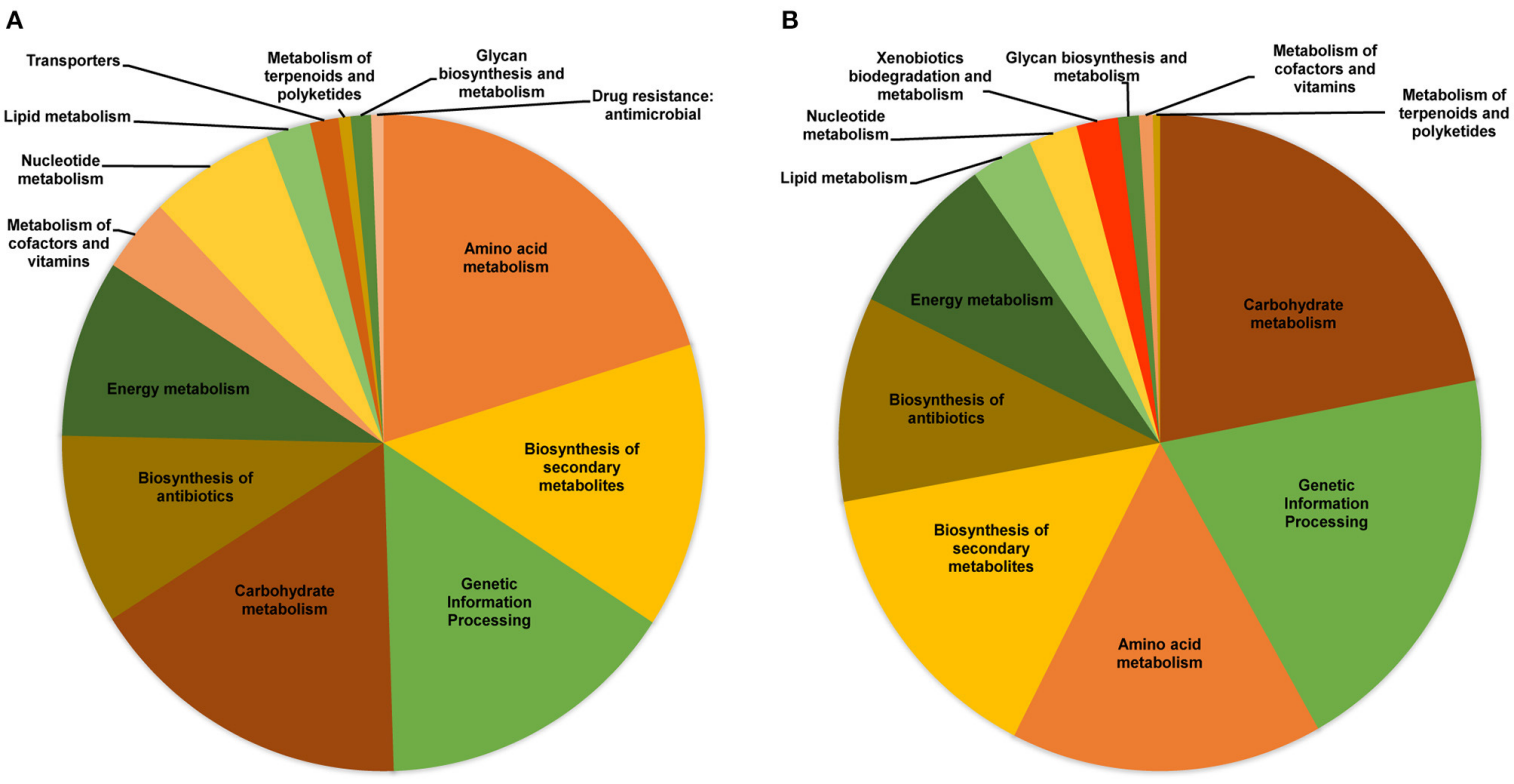
General functional classification base on KEGG annotations of metaproteome data. **(A)** Bacteria proteins. **(B)** Fungi proteins.

Based on the KEGG Orthology Annotation System, a total of 491 proteins for bacteria and 287 proteins for fungi could be annotated to a wide spectrum of metabolic pathways (Figures 5A,B).

For bacteria, proteins associated with amino acid metabolism (20.16%), biosynthesis of secondary metabolites (14.05%), genetic information processing (15.27%) and carbohydrate metabolism (15.27%) were the most abundant (Figure 5A). For fungi, five main processes were found: carbohydrate metabolism (21.95%), genetic information processing (19.86%), amino acid metabolism (15.68%), biosynthesis of secondary metabolites (14.63%) and biosynthesis of antibiotics (10.10%) (Figure 5B). In both cases, proteins associated with energy metabolism, metabolism of cofactors and vitamins, nucleotide metabolism and lipid metabolism were found but in minor abundance. The most relevant differences were observed in proteins related to transport systems and drug resistance present only in bacteria, while proteins for xenobiotic biodegradation and metabolism were present only in fungi.

#### Meta-Analysis of the Metaproteome

**Figure 6:**
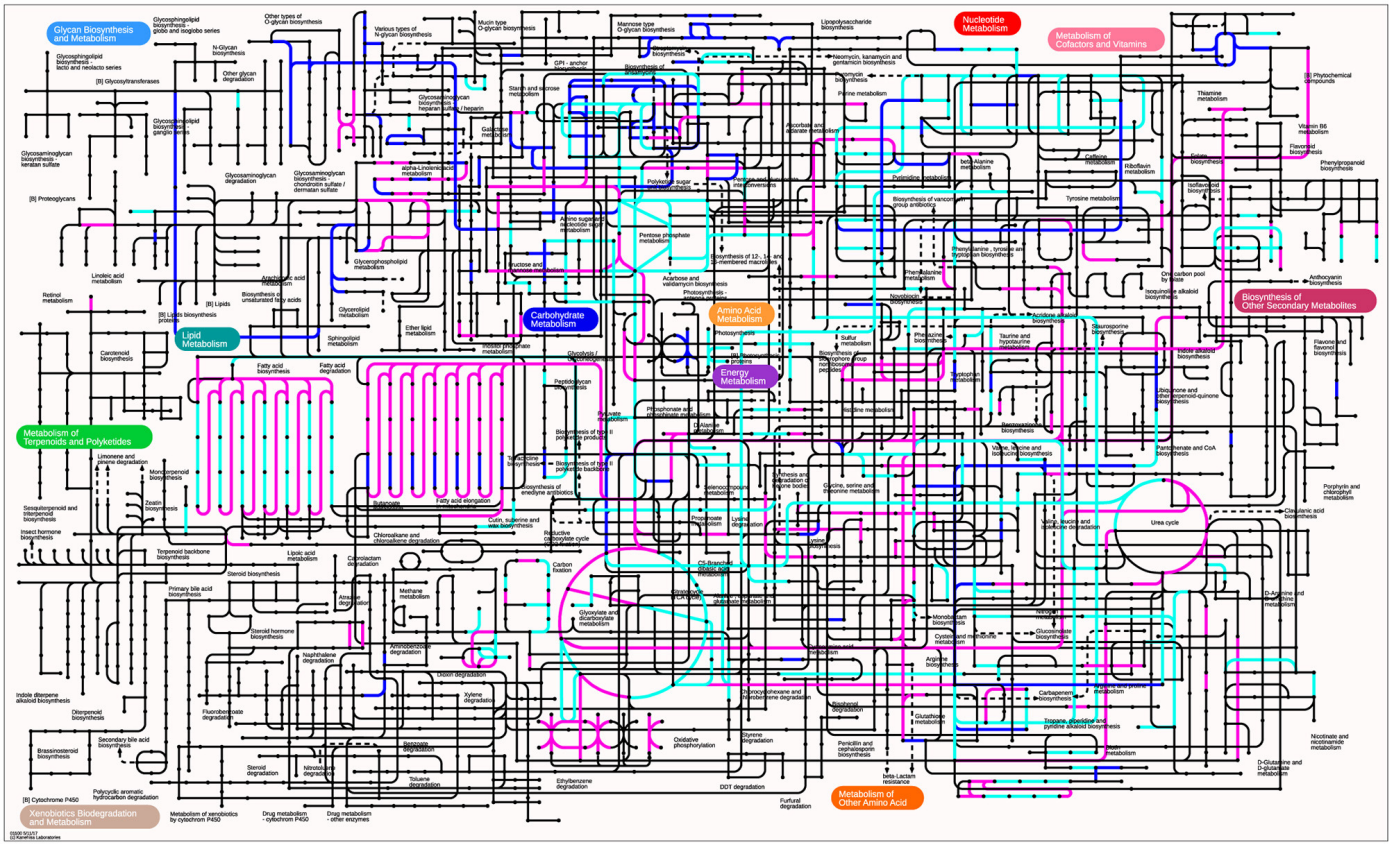
KEGG atlas of the metabolic pathways found in the pozol metaproteome. The proteins identified only in bacteria and fungi are shown in blue. Proteins identified only in plants are shown in pink. The cyan lines indicate common proteins.

The meta-pathways of pozol fermentation were reconstructed based on the metaproteomic data for bacteria, fungi and maize. For each protein, a KO identifier was assigned for its integration into a metabolic pathway. A total of 1102 identifiers were analyzed, resulting in the partial or total reconstruction of different metabolic pathways related to central carbon metabolism, amino acid metabolism, lipid metabolism, synthesis of cofactors and vitamins and energy metabolism (Figure 6).

In the metabolic pathways related to carbohydrate central metabolism, proteins for glycolysis, gluconeogenesis, pyruvate oxidation, Krebs cycle and the pentose phosphate pathway were identified. For amino acid metabolism, different proteins were grouped for biosynthesis and degradation. However, the analysis showed that only in some cases was possible to completely reconstruct the pathways. In lipid metabolism, proteins for fatty acid degradation, fatty acid biosynthesis, long-chain fatty acid elongation, biosynthesis of unsaturated fatty acids, cholesterol and biosynthesis of other lipids (lactosylceramide, triacylglycerol and jasmonic acid) were retrieved. Regarding the biosynthesis of cofactors and vitamins, only a few proteins were identified, and in none of the cases the complete pathways were found.

As expected, proteins related to carbon fixation were found in processes exclusive to plants, such as photosynthesis, to produce energy in the form of ATP and NADPH. Other carbon fixation pathways were the reductive citric acid cycle and the reductive acetyl-CoA pathway.

As shown in Figure 6, the microbial community activities and raw material (maize) enzymes may display metabolic complementation and contribute to the same biosynthetic pathway. However, some metabolic processes were exclusively related to maize or bacteria and fungi. With the metaproteome data, reconstruction of the entire metabolic map of pozol was performed.

##### Carbohydrate Metabolism

*Pyruvate Metabolism* Pyruvate, a key intermediate for flavor compounds production, could be produced in pozol through glycolysis. Pyruvate can be further metabolized in pozol by different mechanisms. First, the enzymes related to the production of acetyl-CoA, which is then metabolized into acetate and ethanol, were retrieved. Second, the reduction of pyruvate to lactate by lactate dehydrogenase was evidenced. Third, pyruvate decarboxylase, which is necessary for acetaldehyde production, was found. Fourth, the analysis showed the presence of the enzyme formate acetyltransferase, which catalyzes the reversible conversion of pyruvate and coenzyme-A into formate and acetyl-CoA. Finally, pyruvate was found to be metabolized to acetoin, diacetyl and 2,3-butanediol via butanoate metabolism (Figure 6, Supplementary Figure 1).

Additionally, the acetyl-CoA from pyruvate and oxaloacetate (produced from aspartate) could be condensed to produce citrate. Although we could not find a transport system for this molecule, the analysis allowed us to identify the enzymes required for the oxidation, decarboxylation and dehydration reactions to form succinate, fumarate and malate.

**Figure 7:**
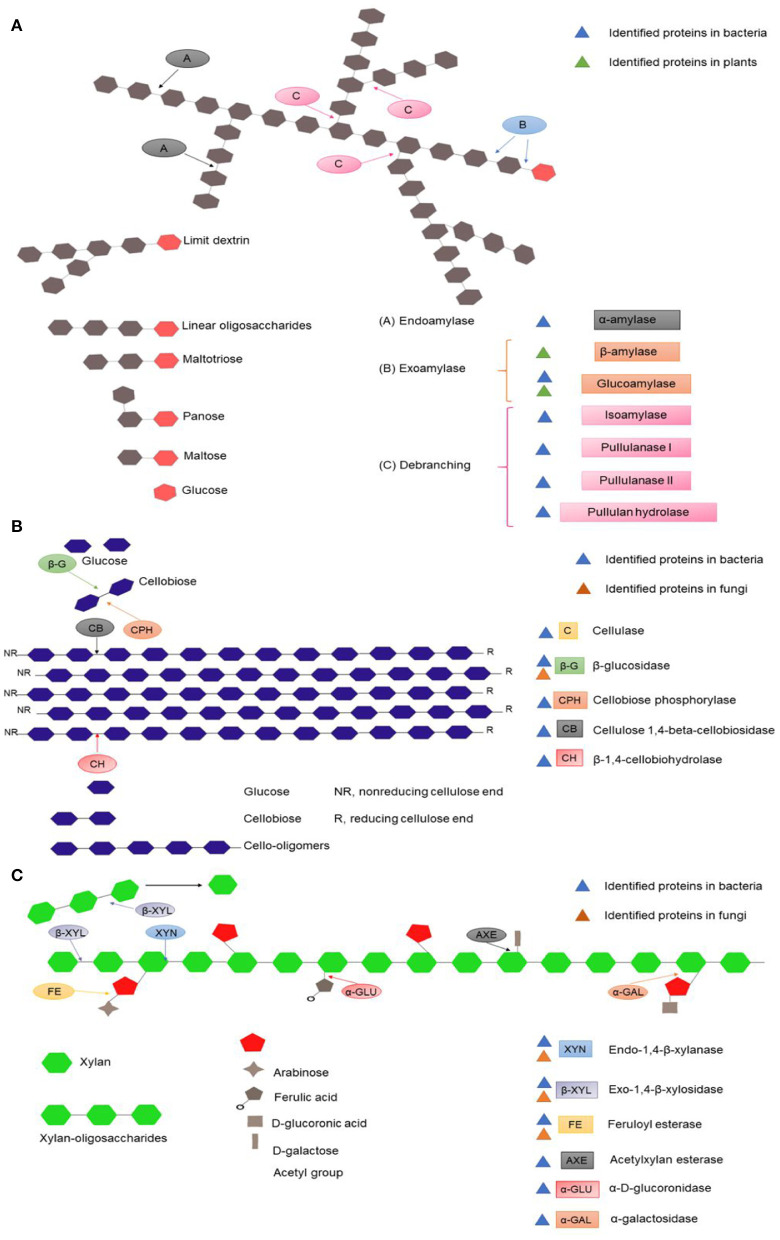
Schematic representation of the different polysaccharides presents in maize dough and the enzymes implicated in their degradation. **(A)** Identified proteins for starch hydrolysis; **(B)** Identified proteins for cellulose hydrolysis; **(C)** Identified proteins for arabinoxylan.

*Polysaccharide Degradation* Several polysaccharides, such as starch, cellulose and hemicellulose, were present in the dough and can be hydrolyzed by the microbiota during fermentation, providing monosaccharides, disaccharides and oligosaccharides, which serve as the most important carbon sources for microbial growth. Thus, a more in-depth analysis was carried out in the search for the enzymes that participate in the metabolism of these carbohydrates (Figure 7; Supplementary Table 1).

Analysis with the CAZy (Carbohydrate Active Enzymes) database allowed the identification of proteins from plants, fungi and bacteria. Most of the proteins were related to bacteria, and the predominant activities were related to starch degradation, while the number of identified proteins was similar for hemicellulose and cellulose. In the case of fungi, fewer proteins were identified, and the majority were involved in cellulose degradation. For plants, two different proteins were identified for starch degradation and one for cellulose degradation.

Analysis with the UniProt database allowed the identification of enzymes for starch, cellulose and hemicellulose degradation, all of which were associated with fungi. Additionally, other glycosyl hydrolases involved in the utilization of other carbohydrates were detected (data not shown).

Transporters for some products of polysaccharide degradation, such as glucose, mannose, galactose, cellobiose and some oligosaccharides (maltooligosaccharides, arabinooligosaccharide and maltodextrin), were also found.

##### Amino acid and Protein Metabolism

Amino acids can be produced from precursors by their metabolism or by the action of different proteases for protein degradation and recycling. In the metaproteomic analysis, the results showed that amino acid biosynthesis occurs by the three mechanisms.

For glutamate, glutamate dehydrogenase was identified as involved in the reductive amination of the intermediary α-ketoglutarate to glutamate. This amino acid is subsequently metabolized to arginine, glutamine and proline. Arginine biosynthetic enzymes from ornithine were identified. The enzyme glutamine synthetase responsible for glutamate and ammonia condensation to form glutamine was also found. The pathway for proline biosynthesis was incomplete; however, proline can be obtained from protein degradation by the action of proline iminopeptidase (Figure 6; Supplementary Figure 2; Supplementary Table 2).

The proteins involved in the metabolism of oxaloacetate for aspartate and asparagine synthesis were identified. Although methionine and threonine are also synthesized from this precursor, the analysis showed that if these amino acids are produced during fermentation, they are produced from homocysteine and homoserine, respectively (Figure 6; Supplementary Figure 2; Supplementary Table 2).

Finally, for the amino acids serine, lysine, tyrosine, histidine, leucine, isoleucine and valine, the proteins for the synthesis of their respective precursors were detected. Nevertheless, in some cases, not all the enzymes necessary to complete the metabolic pathways were identified, or the pathways were truncated in the final step (Figure 6; Supplementary Figure 2).

Concerning amino acid degradation, aminotransferases for the transamination reaction were identified; however, no proteins for the subsequent conversion of α-keto acids were retrieved, indicating that amino acid degradation for flavor production is not a key metabolic pathway during pozol fermentation.

Regarding protein degradation, different proteases, aminopeptidases, and carboxypeptidases were identified, so this can be an alternative pathway to obtain amino acids as well as for the release of peptides of different sizes. Finally, several amino acid, peptide, dipeptide and oligopeptide transport proteins were also detected.

## Discussion

### Proteome Dynamics in Pozol Fermentation

The microbial community in traditional fermented foods has been studied with the use of culture-dependent and culture-independent strategies. Different methods based on DNA analysis (RAPD, AFLP, ARDRA, DGGE) have been commonly used; however, these approaches have inherent biases and do not allow an understanding of the role of microorganisms in fermentation. With the introduction of omics techniques, it is easier to dissect the complexity of microbial-rich environments such as fermentation since microbial community dynamics and functionality can be established. Metaproteomics can suggest the presence of microbial species by assigning identified proteins. This approach allows the study of microbial activity within the system and simultaneously provides functional knowledge about the community under analysis.

The microbiology of pozol has been extensively described (3–9, 15, 22–27) and some of these studies have focused on the description of the dynamics of the microbial community, their spatial distribution and the possible relationship between the chemical changes during fermentation and microbiota development. However, the role of microorganisms during fermentation, their enzymatic activities and therefore their metabolic potential and how they contend with this particular environment are still unknown. In this study, a combination of chemical, culture and metaproteomic analyses was employed to investigate pozol fermentation.

Pozol metaproteomic analysis revealed that this fermented food is a complex ecosystem where bacteria, fungi and archaea are found. The most abundant proteins were identified from the substrate, i.e., more than 60.5% of the identified proteins were from plants; there was an important representation of bacterial and fungal proteins, which accounted for 28.5 and 9.4% of the metaproteome, and only 1.6% of the metaproteome was associated with archaea.

The analysis by fermentation time showed that the unfermented pozol was composed of heterogeneous microbiota. At this point, the identified proteins were related to environmental bacteria, the substrate, the material used for pozol production or skin microbiota. However, these species were reduced or eliminated during fermentation, consequently, it is very likely that they do not participate in the process.

As fermentation took place, a core of microorganisms was established, composed mainly of LAB, whose proteins were identified in high abundance throughout the fermentation. LAB are present in fermented foods prepared from different raw materials, such as milks, cereals, vegetables and animal sources. LAB metabolic activity is closely related to improving the taste, aroma, texture, shelf life, and nutritional value of fermented foods. The production of organic acids (lactic, acetic and formic) by LAB not only impacts the characteristic flavors of the food but also restricts the growth and survival of pathogenic microorganisms by decreasing the pH (28–30). During pozol fermentation, the drastic reduction in pH of 7.75 to 4.83 was associated with the increase in LAB concentration and the number of proteins identified for this group.

After 9 h of fermentation, bacteria of the genus *Streptococcus* dominated the fermentation, in concordance with previous microbiological studies (5–7, 9). *Streptococcus* spp. are widely distributed bacteria in food fermentations, and they have great importance in medicine and industry (31–34). Several streptococci are commensal in mammals; however, some can also cause illnesses ranging from mild to acute. In industry, *Streptococcus thermophilus* is widely used as a yogurt and cheese starter.

Since 2003, Diaz-Ruiz and coworkers (9) described several amylolytic streptococci in pozol, being *S*. *infantarius* subsp. *infantarius*, the most amylolytic. More recently, Cooper et al. (26) established that this same microorganism could grow in corn arabinoxylan. Clearly, the diversity of enzymes that these bacteria express and their adaptability for surviving and developing in extreme pH conditions, both alkaline and acidic (27), allows them to dominate during the early fermentation stages of this dough and persist during the entire fermentation process even when other LAB become more abundant.

Other proteins of lactic acid bacteria identified in the metaproteome belonged to *Lactobacillus, Leuconostoc* and *Enterococcus*, bacteria previously described as present in pozol (4–9, 35). Their presence during fermentation is determined by their capacity to degrade some of the polysaccharides present in the dough. The low abundance of *Enterococcus* during fermentation, compared to the *Lactobacillus* and *Leuconostoc* genera, could be related to their low amylolytic activity (36) and to the fact that some *Lactobacillus* and *Leuconostoc* species can use starch and xylan as the only carbon sources for growth (4, 37, 38). Contrary to what was found in pozol, in other amylaceous fermented foods prepared from cassava, maize and other cereals, *Lactobacillus* is the predominant amylolytic genus in fermentation (39–41), the relatively low abundance of this genus in pozol might be the result of the conditions generated by the nixtamalization process (9).

Other important changes in the microbial composition were observed in the enterobacteria group. Both the plate count and the metaproteomic analysis showed that this group decreased throughout fermentation but was still present even at low pH. Diverse studies have reported the presence of enterobacteria in different stages of the fermentation process (3, 5, 6, 35, 42), and the characterization of these microorganisms has allowed the identification of several serotypes of *Escherichia coli, Escherichia fergusonii, Enterobacter cloacae, Enterobacter aerogenes, Enterobacter sakazakii, Enterobacter* spp., *Klebsiella pneumoniae, Klebsiella* spp., *Klebsiella variicola* and *Kosakonia* spp (1, 15, 43, 44). Their presence even at high organic acid concentrations has been attributed to the existence of microenvironments within the dough, to the acid tolerance of some strains or to the use of organic acids by other microorganisms for their growth, decreasing the local concentration (6, 7, 44, 45). Despite their negative reputation, some of these bacteria can directly fix atmospheric nitrogen in the dough, which allows for its nutritional enrichment (15).

Throughout fermentation, fungal proteins were identified, mainly represented by yeast proteins, both in the unfermented dough and in the different fermented samples, with an increase in the number of identified proteins of filamentous fungi at the end of fermentation. The presence of yeasts since the first hours of fermentation is related to their short reproductive cycles, while filamentous fungi developed when the surface dried and pH decreased, while yeasts continued to grow (46, 47).

Proteins of the filamentous fungi *Ajellomyces, Cercospora* and *Fusarium* were identified only in the unfermented dough, which could indicate that these species do not survive the changes that are generated during fermentation or that the substrate does not have the necessary conditions for their development. In the fermented samples, the predominant genera were *Schizosaccharomyces, Saccharomyces* and *Neurospora*.

Proteins of other genera, such as *Aspergillus, Penicillium, Puccinia, Kluyveromyces* and *Komagataella*, showed fluctuating changes throughout fermentation. The development of this group was associated with the production of organic acids by LAB. It has been suggested that the proliferation of fungi in foods is stimulated by the acidic environment that LAB generate by their metabolism, and in turn, fungi favor the growth of other bacteria, providing growth factors such as vitamins and soluble nitrogen compounds (48).

These genera have been reported in other fermented foods based on wheat or rice flour or wheat or barley grains as well as in traditional alcoholic beverages and fermented milk products (49–51). Although fungi in fermented foods can be considered harmful due to the production of enterotoxins, some of them may have an important role during the fermentation process due to the production of enzymes for the degradation of complex polysaccharides and antinutritional factors. Additionally, the metabolism of these organisms can result in an improvement in the organoleptic characteristics and nutritional value through the production of characteristic flavors due to the degradation of fatty acids and the production of vitamins, amino acids and folic acid (52–56).

### Microbial Metabolism in Pozol

#### Degradation of Polysaccharides

Pozol is characterized by a low concentration of soluble carbohydrates and a high starch content that must support an abundant microbiota. ALAB have been considered the most relevant group during fermentation to provide glucose and maltooligosaccharides for the nonamylolytic microbial community. Although ALAB are present at high concentrations (10^8^ CFU · g of dry weight ^−1^), its low amylolytic activity does not explain the rich microbial diversity and the high abundance of nonamylolytic microorganisms (9). In addition, it should be considered that in nixtamal dough, alternative carbon sources, hemicellulose and cellulose, are available for fermentation.

For starch hydrolysis, two proteins from maize were identified: a β-amylase that produces maltose and an α-glucosidase that produces glucose from starch and maltose. These enzymes could play an important role in pozol, mainly in the first stage of fermentation, releasing simple sugars that can be used for the initial growth of microorganisms. In bacteria, multiple amylolytic enzymes, exoamylases (α-amylase) and endoamylases (α-glucosidase), and debranching enzymes (pullulanase), were identified. In combination, these enzymes allow the depolymerization of starch, demonstrating the importance of this group during fermentation (Figure 7A). Previous studies demonstrated the presence of amylolytic bacteria and yeast (4, 9); however, no yeast amylase was identified in the metaproteome. Characterization of the most amylolytic LAB species, *Streptococcus infantarius* subsp. *infantarius*, produced two amylases, an amylopululanase with debranching capacity and an α-amylase (9, 57).

On the other hand, the pericarp is a source of crude fiber and consequently an important source of carbon. The fiber in maize kernels has been reported to contain ~40% arabinoxylan and 20% cellulose (58). However, their degradation requires the action of several enzymes for the hydrolysis of the backbone and the different residues that they may contain.

Corn arabinoxylan is formed by a main chain of d-xylopyranose linked in β- (1,4) with side chains of α-1-arabinofuranose. A high degree of structural heterogeneity is given by other sugars in the branches, including galactose, glucuronic acid and xylose (59). Metaproteome analysis showed that in pozol, bacteria and fungi might degrade hemicellulose into xylose, xylobiose and xylo-oligosaccharides, since the enzymes for the degradation of the main chain and the different substitutions were identified (Figure 7C). Previous studies have demonstrated that bacteria isolated from pozol are able to grow and metabolize xylan and arabinoxylan (26).

Cellulose degradation requires three classes of enzymes: β-1,4-endoglucanases (EGL), exoglucanases/cellobiohydrolases (CBH), and β-glucosidase (BGL). Endoglucanases from bacteria and plants were found in the metaproteome, and this enzyme is necessary to initiate the hydrolysis of the polysaccharide, reducing the degree of polymerization (60). In addition, enzymes for the production of cellobiose, glucose and cello-oligosaccharides from cellulose were identified (Figure 7B).

Together, the metaproteome data and chemical analysis demonstrate that during fermentation, starch, hemicellulose and cellulose are fermentable carbon sources. Changes in the carbohydrate content can be associated with the enzymatic activities identified for the degradation of the different polysaccharides. During the first 9 h of fermentation, the crude fiber concentration significantly decreased, indicating that in this period, the microbiota consumes polysaccharides such as cellulose and hemicellulose for the release of simple carbohydrates, which in fact increased (Table 1). Finally, the analysis of proteins suggests a possible synergism between bacteria, fungi and plants for the degradation of the different substrates.

#### Lipid Metabolism

During carbohydrate catabolism through glycolysis, acetyl-CoA is produced, and its main function is energy production in the TCA cycle and as an intermediate in fatty acid, leucine and lysine biosynthesis.

Fatty acid synthesis starts with acetyl-CoA carboxylation to malonyl CoA, in the pozol metaproteome, acetyl-CoA carboxylase was present. The set of enzymes for the subsequent series of condensation, reduction and dehydration reactions included fatty acid synthase (FAS) enzymes. In the analysis, two different types of FASs were identified, type I and type II. FAS type I multienzymes are found in eukaryotic organisms (animals and fungi) and in a few bacteria, and the FAS type II system occurs in archaea, prokaryotic organisms and plastids of plants (61). This set of enzymes allows the production of fatty acids with different chain lengths and the generation of unsaturated or saturated fatty acids (62, 63). These molecules have fundamental roles as the main constituent of cellular membranes, where they form the precursors of phospholipids, sphingolipids and sterols as secondary metabolites and signaling molecules, representing a suitable compound for energy and carbon storage. Consistent with the presence of enzymes for fatty acid biosynthesis, an increase in lipid concentration in the dough was observed (Table 1).

Additionally, enzymes for fatty acid degradation to produce acetyl-CoA were found. Acetyl-CoA is oxidized for energy production in the TCA cycle and can be used for conversion to succinate in the glyoxylate cycle for subsequent carbohydrate synthesis. These processes allow microorganisms to synthetize complex molecules that require and utilize simple carbon compounds in the absence of available carbohydrates such as glucose during growth.

### Metabolic Potential of Pozol Microbiota

Fermented foods result from chemical changes in the substrate due to the metabolism of the microorganisms that develop during the process. These microorganisms can modify the raw material in different ways, improving the organoleptic and nutritional properties of the final product. Moreover, during fermentation, microorganisms associated with fermented food or beverages develop multiple functional properties that can stimulate consumer health (64, 65).

#### Flavor Development

As shown in Figure 6 and Supplementary Figure 1, pyruvate metabolism may contribute to the organoleptic characteristics of pozol through three metabolic mechanisms. First, pyruvate is converted to lactate by lactate dehydrogenase. Second, acetyl-CoA is produced and then metabolized to acetate and ethanol. Third, pyruvate is metabolized by acetolactate decarboxylase and by diacetyl reductase to acetoin and 2,3-butanediol. The results imply that in fermentation, homofermentative and heterofermentative metabolism occurs. A previous study by DGGE analysis showed that fermentation can be divided into two stages; in the initial stage, homofermentative bacteria such as *Streptococcus* and *Enterococcus* are present; in the second stage (24 to 48 h), in addition to homofermentative bacteria, heterofermentative LAB, including *L*. *fermentum* and *Leuconostoc* species, develop and reach maximum levels at 48 h. These bacterial activities result in the production of lactate as the major fermentation product, with acetate and ethanol at lower concentrations (7). Although acetoin and butanediol have not been reported in pozol, metaproteomic analysis identified the enzymes involved in their biosynthesis. In other fermented foods, acetoin and butanediol production is mainly associated with LAB metabolism, where these compounds can make a great contribution to flavor (66–68). These results demonstrate the metabolic potential of microbiota to produce a wide range of compounds important for food safety, sensory attributes and the presence of enzymes that may have important biotechnological applications.

#### Nutritional Improvement

Fermentation provides an easy and economical method to enrich the different substrates used for production. During fermentation, different molecules (essential amino acids, vitamins, bioactive peptides, etc.) can be synthesized by microorganisms. The metabolism of microorganism may increase mineral bioavailability, improve protein digestibility, and the degradation of complex macromolecules into simple biomolecules occurs (69, 70).

In pozol, the fermentation of maize dough produces changes in the amino acid and vitamin concentrations. Amino acids such as histidine, leucine, methionine and phenylalanine decreased in the fermented dough; in contrast, the content of other amino acids (arginine, lysine, isoleucine, threonine and tryptophan) increased during fermentation (71). In the metaproteome, several enzymes for amino acid metabolism were grouped in the general classification, and a detailed analysis showed that the synthesis of glutamate, glutamine, serine, arginine, aspartate and asparagine is possible since all the enzymes involved in their respective metabolic pathways were identified. For the rest of the amino acids, none or only some of the required enzymes for their biosynthesis were identified. However, protein degradation through the action of different proteases could release different amino acids, as in the case of proline, which could explain the increments in the essential amino acid concentration. Regarding vitamins, the same study showed that the raw material is enriched during fermentation with riboflavin and niacin, but in the metaproteomic data, fewer proteins were identified for the metabolism of vitamins, being impossible to complete the respective pathways.

## Conclusions

The simultaneous analysis of the microbiology, biochemistry and metaproteomics of pozol allowed the construction of a comprehensive image of the fermentation system. Bacteria, fungi, yeasts and archaea were found to participate in the pozol fermentation process, in addition to the enzymatic systems of the substrate itself, being bacteria the most represented group throughout fermentation.

The main changes both in the substrate and the microbiota occur in the first 9 h of fermentation, and the logarithmic increase in microorganisms is correlated with the decrease in pH and in the content of carbohydrates and fiber, which indicates that this stage has the highest metabolic activity. Many proteins from environmental microorganisms related to corn and water were found for the first time; however, most of them disappeared or drastically decreased over fermentation.

Bacteria were mostly represented by LAB proteins, being the genus *Streptococcus* by far, the most abundant. All these bacteria can be found in the environment, but their most important habitat is in mammals, which indicates that although spontaneous fermentation occurs, human participation in the nixtamalization and production process is decisive in the microbiota that develops. Yeast proteins were present in all the analyzed samples, with an increase in proteins from filamentous fungi at the end of fermentation.

The metaproteomic approach allowed the identification of several unknown proteins in the system so far. Systems for the hydrolysis of starch, hemicellulose and cellulose were also found, indicating that all these substrates can be used as a carbon source by the microbiota. It is striking that nonfungal enzymes were found for starch degradation, and the enzymes from corn and bacteria were responsible for the hydrolysis of this substrate. For hemicellulose and cellulose, both bacteria and fungi were involved, always with a predominance of bacterial enzymes. This diverse repertoire of enzymes found will allow new biochemical and structural studies.

The metabolic process revealed the synthesis of various fermentation products, such as organic acids, acetoin, butanediol and important intermediates, involved in the synthesis of fatty acids and amino acids and the generation of compounds that contribute to the organoleptic characteristics of pozol.

## Data Availability Statement

The datasets presented in this study can be found in online repositories (72, 73). The names of the repository/repositories and accession numbers can be found below: ProteomeXchange Consortium via the PRIDE: Project accession: PXD026648, Project doi: 10.6019/PXD026648, Project accession: PXD026705, Project doi: 10.6019/PXD026705, Project accession: PXD026781, Project doi: 10.6019/PXD026781.

## Author Contributions

RR-S designed the experiments and gave general supervision, CW, SE, and SS contributed to conception and design of the study. JR carried out most of the experimental work, data analysis and wrote the first manuscript. DG and GD-R carried out some of the experimental work. All authors contributed to manuscript revision, read, and approved the submitted version.

## Conflict of Interest

The authors declare that the research was conducted in the absence of any commercial or financial relationships that could be construed as a potential conflict of interest.

## Publisher's Note

All claims expressed in this article are solely those of the authors and do not necessarily represent those of their affiliated organizations, or those of the publisher, the editors and the reviewers. Any product that may be evaluated in this article, or claim that may be made by its manufacturer, is not guaranteed or endorsed by the publisher.
