# Supplementary material for: Metaproteomic Insights Into the Microbial Community in Pozol
Source: Front Nutr. 2021 Aug 20;8:714814. doi: 10.3389/fnut.2021.714814 (PMC8417691; doi:10.3389/fnut.2021.714814)
Supplement: Supplementary file 1 [file Data_Sheet_1.docx]

Supplementary Material

**
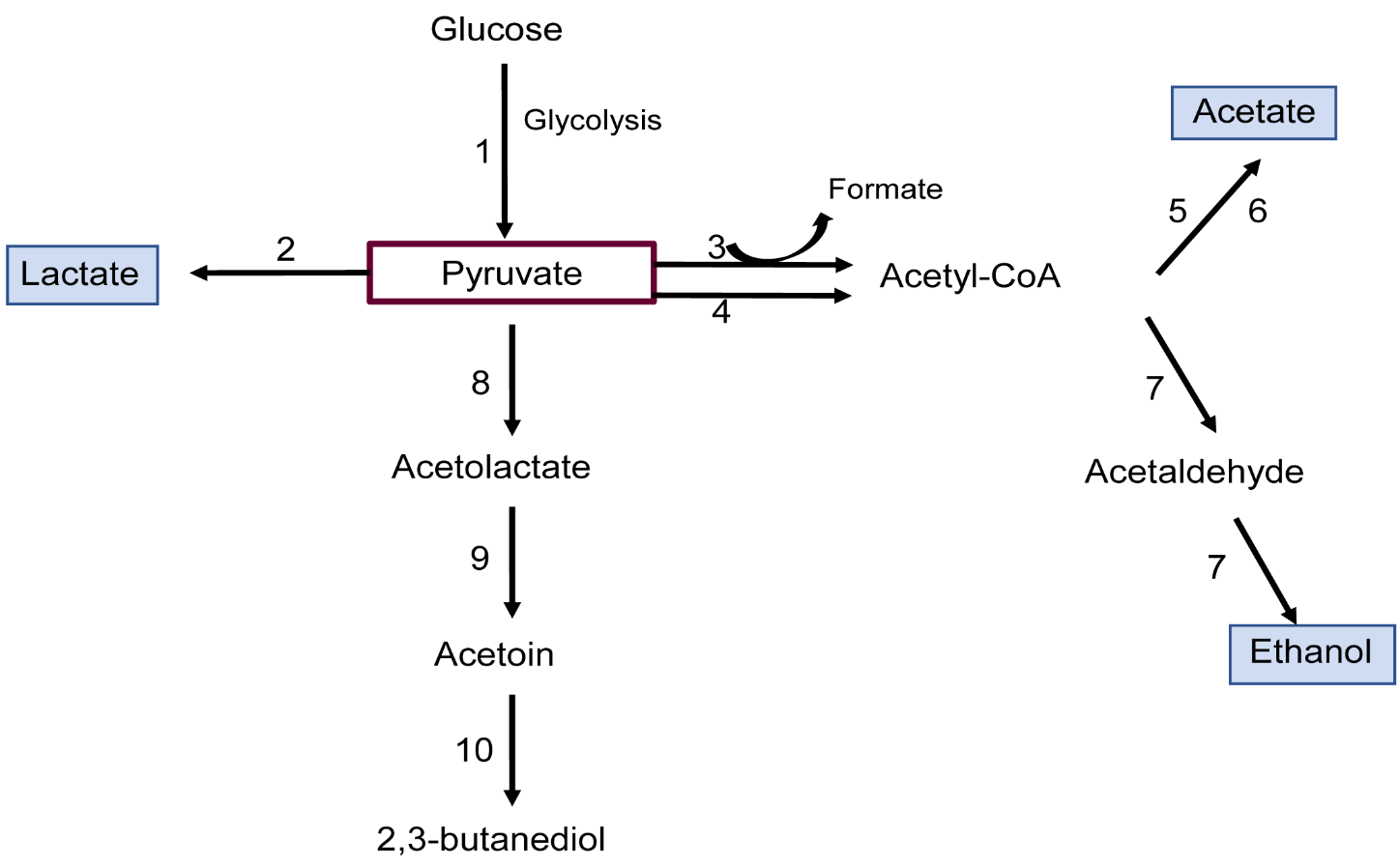
**

**Supplementary Figure 1.** Identified enzymes related to the flavor and odors production in pozol fermentation. 1) Oxaloacetate decarboxylase. 2) Lactate dehydrogenase. 3) Pyruvate formate-lyase. 4) Pyruvate dehydrogenase. 5) Phosphotransacetylase. 6) Acetate kinase. 7) Alcohol dehydrogenase. 8) Acetolactate synthase. 9) Acetolactate decarboxylase. 10) Diacetyl reductase.


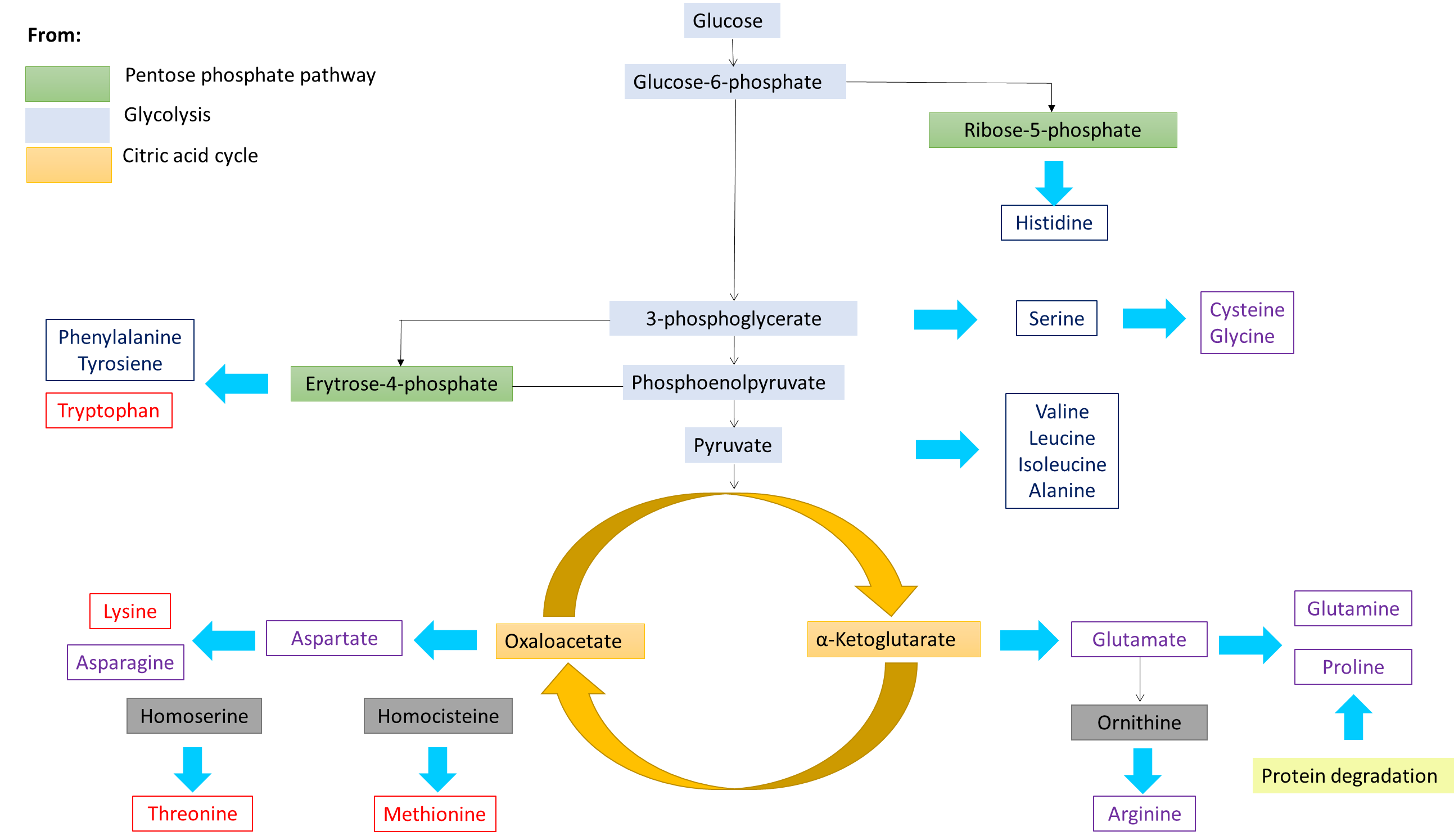


**Supplementary Figure 2.** Amino acid biosynthesis overview. The metabolic pathway and their respective intermediates for amino acids biosynthesis are shown. In the purple square the amino acids that can be produced in the pozol fermentation are shown. In the red square the amino acids for which some of the proteins were identified are shown. In the blue square the amino acids for which the enzymes for their biosynthesis were not identified are shown.

**Supplementary Table 1**. Identified enzymes in the metaproteome for the degradation of the different polysaccharides.

| Substrate | Conventional name | E.C. number | Database/organism |
| --- | --- | --- | --- |
| Starch | Alpha-1,4-glucosidase | 3.2.1.20 | CAZy/Bacteria  CAZy/Plants |
|  | Alpha amylase | 3.2.1.1 | CAZy/Bacteria |
|  | Glucan 1,4-α-glucosidase | 3.2.1.3 |  |
|  | Isoamylase | 3.2.1.68 |  |
|  | Amylopullulanase | 3.2.1.1/41 |  |
|  | Pullulanase | 3.2.1.41 |  |
|  | Neopullulanase | 3.2.1.135 |  |
|  | Beta-amylase | 3.2.1.2 | CAZy/Plants |
|  | | | |
| Cellulose | Beta-glucosidase | 3.2.1.21 | CAZy/Bacteria  CAZy/Fungi  UniProt/ Fungi |
|  | Beta-1,4-endoglucanase | 3.2.1.4 | CAZy/Bacteria  UniProt/ Fungi CAZy/Plants |
|  | Cellobiose phosphorylase | 3.4.1.20 | CAZy/Bacteria |
|  | Cellulose 1,4-beta-cellobiosidase | 3.2.1.91 |  |
|  | | | |
| Hemicellulose | Enzymes involved in the degradation of the backbone | | |
|  | Xylan 1,4-beta-xylosidase | 3.2.1.37 | CAZy/Bacteria  UniProt/ Fungi |
|  | Endo-1,4-beta-xylanase | 3.2.1.8 | CAZy/Bacteria  CAZy/Fungi |
|  | Xyloglucan exo-beta-1,4-glucanase | 3.2.1.155 | CAZy/Bacteria |
|  | Accessory, side-group-removing enzymes | | |
|  | Alpha-L-arabinofuranosidase | 3.2.1.55 | CAZy/Bacteria  CAZy/Fungi  UniProt/ Fungi |
|  | Feruloyl esterase | 3.1.1.73 | CAZy/Bacteria  UniProt/ Fungi |
|  | Xylan alpha-1,2- glucuronosidase | 3.2.1.131 | CAZy/Bacteria |
|  | Alpha-galactosidase | 3.2.1.22 |  |
|  | Acetylxylan esterase | 3.1.1.72 |  |
|  | Alpha-fucosidase | 3.2.1.51 |  |

**Supplementary Table 2**. Identified enzymes in the metaproteome for the synthesis of the different amino acids. All the proteins were identified in the UniProt database.

| Mechanisms | Metabolic pathway | Intermediate | Reaction | Enzyme | Organism |
| --- | --- | --- | --- | --- | --- |
| Biosynthesis | Citric acid cycle | Oxaloacetate | Oxaloacetate Aspartate | Aspartate aminotransferase | Plants  Fungi |
|  |  |  | Saccharopine Lysine | Saccharopine dehydrogenase | Fungi |
|  |  |  | 2-Oxoglutarate Glutamate | Aspartate aminotransferase | Plants  Fungi |
|  |  | α-ketoglutarate | α-ketoglutarate Glutamate | Glutamate dehydrogenase | Bacteria |
|  | Pentose phosphate | Erytrose-4-phosphate | Indole glycerol phosphate Tryptophan | Tryptophan synthase | Bacteria  Fungi |
|  | Urea cycle | Ornithine | L-Arginosuccinate Arginine | Argininosuccinate synthase | Plants  Bacteria |
|  | Sulfur metabolism | Homoserine | O-Phospho-L-homoserine Threonine | Threonine synthase | Plants |
|  |  | Homocysteine | Homocysteine Methionine | 5-methyltetrahydrofolate--homocysteine methyltransferase | Plants  Bacteria |
| Amino acid degradation | Aspartate degradation | NA | Aspartate Asparagine | Aspartate--ammonia ligase | Bacteria |
|  | Glycine degradation |  | Glycine Serine | Glycine hydroxymethyltransferase | Plants  Bacteria  Fungi |
|  | Serine degradation |  | Serine Glycine | Serine hydroxymethyltransferase | Plants  Bacteria  Fungi |
|  | Serine degradation |  | Actetyl-serine Cysteine | Cysteine synthase | Plants |
|  | Glutamate degradation |  | Glutamate Glutamine | Glutamine synthetase | Plants  Bacteria  Fungi |
| Protein degradation | NA | NA | Peptide cleavage and various proteins in an ATP-dependent process | ATP-dependent Clp protease | Plants  Bacteria |
|  |  |  | Peptide cleavage with [histidine](https://en.wikipedia.org/wiki/Histidine), [glutamate](https://en.wikipedia.org/wiki/Glutamate), [aspartate](https://en.wikipedia.org/wiki/Aspartate), [lysine](https://en.wikipedia.org/wiki/Lysine), and [arginine](https://en.wikipedia.org/wiki/Arginine) | ATP-dependent zinc metalloprotease | Bacteria |
|  |  |  |  | Probable metalloprotease | Fungi |
|  |  |  | Cleaves proteins in the C-terminal | Cysteine protease | Plants  Fungi |
|  |  |  | Family of proteases that cleave the dipeptide bonds that have hydrophobic residues | Aspartic proteinase | Plants |
|  |  |  | Cleaves a wide spectrum of amino acid substrates and several peptide substrates | Proline iminopeptidase | Bacteria |
|  |  |  | Degrades polypeptides processively to yield small peptide fragments that are 5 to 10 amino acids long | ATP-dependent serine peptidases | Bacteria  Fungi |
|  |  |  | Hydrolyzes the peptide bond at the imino side of aromatic residues. | Neutral protease | Fungi |
|  |  |  | Cleaves of proteins and peptides | Peptidase beta subunit | Plants |
| Protein recycling | NA | NA | Peptide cleavage with arginine, phenylalanine, tyrosine, leucine and, glutamic acid | Proteasome | Plants  Fungi |

NA: Indicates those processes that do not occur as part of a metabolic pathway or don have intermediaries.

**
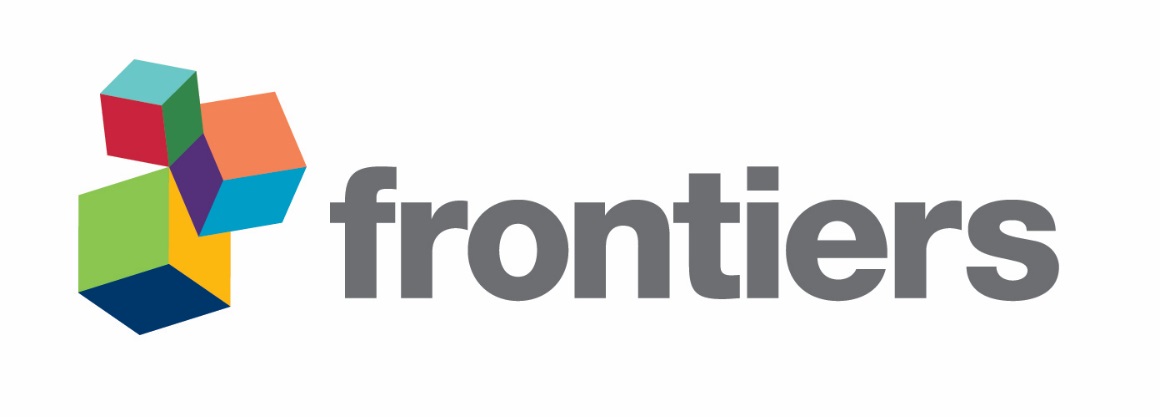
**
